# No associations between HIV reservoir and inflammation in long-term virally suppressed dolutegravir-based ART-treated individuals

**DOI:** 10.3389/fimmu.2025.1628086

**Published:** 2025-07-28

**Authors:** Céline Fombellida-Lopez, Diego Aguilar Ortmans, Michel Moutschen, Alexander O. Pasternak, Gilles Darcis

**Affiliations:** ^1^ Immunology and Infectious Diseases Laboratory, Grappe Interdisciplinaire de Génoprotéomique Appliquée (GIGA)-Institute, University of Liège, Liège, Belgium; ^2^ General Internal Medicine and Infectious Diseases Department, University Hospital of Liège, Liège, Belgium; ^3^ Laboratory of Experimental Virology, Department of Medical Microbiology, Amsterdam University Medical Center (UMC), University of Amsterdam, Amsterdam, Netherlands

**Keywords:** HIV reservoir, inflammation, immune activation, correlation, antiretroviral therapy

## Abstract

**Introduction:**

Despite effective antiretroviral therapy (ART), people with HIV (PWH) experience persistent immune activation and inflammation, increasing the risk of non-AIDS-related comorbidities. The contribution of the HIV reservoir to this chronic inflammatory state remains debated. Understanding the relationship between HIV persistence, immune activation, and inflammation is crucial for optimizing long-term therapeutic strategies.

**Methods:**

This study assessed HIV persistence, immune activation, and systemic inflammation in 49 PWH treated with the same dolutegravir-based triple ART regimen. HIV reservoir size and activity were evaluated by measuring total HIV DNA in peripheral blood mononuclear cells (PBMCs) and rectal tissue, cell-associated (CA) unspliced (US) HIV RNA, and residual viremia. Over 20 inflammatory biomarkers, including sCD14, IL-6, TNF-α, and CXCL10, were analyzed, along with comprehensive immune profiling using a 26-color spectral flow cytometry panel. Clinical parameters such as age, nadir CD4 count, and co-infections were also considered.

**Results and discussion:**

Our findings showed a limited association between HIV persistence markers and systemic inflammation or immune activation. Compared to previous studies, participants had lower reservoir sizes and transcriptional activity, likely due to early ART initiation and prolonged suppression. Immune preservation was evident, with high CD4/CD8 ratios and reduced activation markers. These results challenge the idea that the HIV reservoir is the primary driver of chronic inflammation in PWH on a dolutegravir-based long-term ART. Instead, the reservoir may evolve toward a more transcriptionally silent and defective state, reducing its impact on systemic immune activation.

## 1 Introduction

Human immunodeficiency virus (HIV) infection remains a major global health issue, affecting an estimated 39.9 million people in 2023 (WHO, HIV and AIDS, Fact sheets, 22 July 2024 (1)). Antiretroviral therapy (ART) has transformed HIV infection into a manageable chronic condition and brought the life expectancy of people living with HIV (PWH) nearly in line with that of uninfected individuals (2, 3). However, ART is not curative and requires life-long adherence; while it effectively suppresses viral replication, it does not eradicate the virus from latent reservoirs or fully restore immune function (4–6). PWH often suffer from chronic immune activation and inflammation, increasing their risk of non-AIDS-related comorbidities, such as cardiovascular diseases, neurocognitive disorders, and metabolic syndrome (7, 8).

This persistent inflammatory state in ART-treated PWH is complex and multifactorial (9). HIV persists in viral reservoirs, especially within CD4+ T cells and monocytes (10, 11), where it can produce low levels of viral RNA, and even viral proteins, potentially triggering antiviral immune responses (12). Additionally, low-level viral replication in sanctuary sites, where ART do not fully penetrate, may contribute to ongoing inflammation (13, 14). Microbial translocation, which is the leakage of bacterial products from the gut into the bloodstream due to gut-associated lymphoid tissue (GALT) disruption by HIV, further stimulates immune responses and increases levels of inflammatory mediators, such as lipopolysaccharide (LPS) and soluble CD14 (sCD14) (15, 16). Microbial translocation is linked to thymic function failure, another crucial factor involved in HIV disease progression and immune dysregulation (17). Immune dysregulation, particularly in CD4+ and CD8+ T cells, adds to this pro-inflammatory environment. CD4+ T cell depletion drives the release of inflammatory cytokines (e.g., IL-6, TNF-α), which are closely associated with poorer clinical outcomes in individuals with chronic HIV infection (18). Moreover, ART-treated individuals often show increased immune exhaustion (PD-1, LAG-3) and activation markers (CD38, HLA-DR) on T cells. These markers correlate with larger HIV reservoirs and reduced T-cell function (19, 20). Monocytes and other immune cells also contribute significantly to the inflammatory milieu (21). Monocytes play a central role in sustaining chronic inflammation, as they release pro-inflammatory cytokines and chemokines, which further activate immune pathways (22).

Lastly, clinical factors like age, gender, nadir CD4 count, co-infections (e.g. CMV, hepatitis B, hepatitis C, EBV), and timing of ART initiation, also influence inflammation levels. For instance, older age is associated with poorer immune responses (23–25), a phenomenon increasingly referred to as “inflammageing”, which has become more evident as ART extends life expectancy in PWH (26). Similarly, gender differences have been noted, with the female gender being linked to higher levels of immune activation (27, 28).

Understanding the relationship between HIV persistence markers and immune activation is crucial, as the inflammatory environment not only drives T-cell proliferation but also perpetuates a vicious cycle that maintains and replenishes viral reservoirs (13).

In this study, we aim to investigate the associations between HIV persistence, inflammatory markers, clinical indicators, and immune cell populations in a cohort of 49 PWH treated with the same dolutegravir-based ART regimen. We analysed over 40 parameters, categorized into four main groups:

1. HIV Persistence Markers: Total HIV DNA in PBMCs and in rectal biopsies (GALT), cell-associated (CA) unspliced (US) HIV RNA in PBMCs, and residual viremia.
2. Plasma inflammatory Markers: A panel of more than 20 cytokines, chemokines, and cell adhesion/inflammatory response molecules, including sCD14, High-sensitivity CRP (hs-CRP), IL-6, TNF-α, IFN-γ, CXCL10, and ICAM-1.
3. Immune Cell Populations: Using a 26-color spectral flow cytometry panel, we performed immunophenotyping to identify various immune cell populations, including T cells, B cells, monocytes, NK cells, regulatory T cells (Tregs), mucosal-associated invariant T (MAIT) cells, gamma delta (γδ) T cells and innate lymphoid cells (ILCs). We also assessed markers of immune activation (HLA-DR and CD38) and immune exhaustion (PD-1 and TIGIT).
4. Clinical Indicators: Patient demographic and health data, including gender, age, BMI, smoking status, ethnicity, nadir CD4 count, CD4/CD8 ratio, pre-ART zenith plasma viral load, time to treatment (the period between diagnosis of HIV infection and the start of treatment) and time of suppressive ART (duration between the initiation of the current suppressive ART regimen and the first study visit).

This research seeks to deepen our understanding of the complex immune landscape in ART-treated PWH and its implications for managing HIV-associated inflammation and related comorbidities.

## 2 Materials and methods

### 2.1 Study design and participants

This study combined the baseline data from two open-label, interventional, monocentric, randomized, and controlled clinical trials conducted at the University Hospital of Liège, Belgium, involving a total of 49 HIV-1-infected adults on long-term ART. Both trials focused on individuals treated with a combination of dolutegravir (DTG, 50 mg), abacavir (ABC, 600 mg), and lamivudine (3TC, 300 mg) for at least two years, with sustained viral suppression.

In the first study (ClinicalTrials.gov identifier: NCT05351684), 13 participants were enrolled in a phase 2 trial assessing the impact of DTG intensification (an additional daily dose of 50 mg) compared to a control group that continued the standard DTG/ABC/3TC regimen. Eligibility criteria included a consistently suppressed plasma viral load below 20 HIV RNA copies/mL for at least 12 months prior to screening (allowing brief “blips”) and an absolute CD4+ T lymphocyte count above 200 cells/mm³. Ethics approval was granted by the Liège University Hospital-Faculty Ethics Committee (2018/228), and all participants provided informed consent. The second study (ClinicalTrials.gov identifier: NCT04034862) was a phase 3 trial involving 36 participants. This trial aimed to evaluate the effects of treatment simplification to dual therapy (50 mg DTG + 300 mg 3TC) versus continued triple therapy. Inclusion criteria were identical to the first study: a viral load consistently below 20 HIV-1 RNA copies/mL for the previous 12 months and a CD4+ T cell count above 200 cells/mm³. Ethics approval for this study was provided by the same ethics committee (2018/292), and each participant provided written informed consent.

Detailed inclusion and exclusion criteria for both studies are available in **Supplementary Data 1 and 2**.

### 2.2 Methods

All methods used for the quantification of HIV persistence markers (including total HIV DNA in PBMCs and rectal biopsies, cell-associated HIV RNA in PBMCs, intact HIV DNA in PBMCs, and residual viremia), plasma inflammatory biomarkers, and immune cell populations are detailed in the original studies from which the data were derived (29, 30).

### 2.3 Statistical analysis

Statistical analyses were performed using R (version 4.2.2) and R studio (version 2023.6.1.524). Differences were tested for statistical significance using Mann-Whitney U tests for continuous variables and Fisher’s exact tests for categorical variables. Spearman’s rank method was used to determine the correlation coefficients. Correlograms were generated using the “corrplot” R package (version 0.95). For some variables, certain values were above or below a detection threshold set by the measuring instrument. For values below this threshold, a value between 0 and the threshold was randomly determined from a triangular distribution whose mode corresponds to the threshold value divided by 2. For values above a maximum detection threshold, the value of this threshold was used.

## 3 Results

### 3.1 Study design and participants

Forty-nine ART-suppressed participants, all receiving the same triple antiretroviral regimen consisting of 50 mg dolutegravir (DTG), 600 mg abacavir (ABC), and 300 mg lamivudine (3TC) for at least two years, were enrolled from two separate clinical trials (EDIT, NCT05351684 and IDOLTIB, NCT04034862) conducted at the University Hospital of Liège. The cohort consisted of thirteen participants from the EDIT study and thirty-six from the IDOLTIB study, all maintaining continuous suppressive treatment for a median of 7.62 years.

The baseline clinical characteristics of this combined cohort are presented in **Table 1**.

**Table 1:** Clinical characteristics of participants at baseline.

	All (n=49)	EDIT (n=13)	IDOLTIB (n=36)	Pa
Sex, male	42 (85.71)	13 (100)	29 (80.56)	0.1668
Age, years	48 (40-60)	51 (46-56)	48 (39-60)	0.6585
BMI, kg/m²	24 (22-28) N = 48	22 (21-25)	25 (23-28) N = 35	0.05755
Ethnicity				0.02865
Caucasian	34 (69.39)	11 (84.62)	23 (63.89)	
African	14 (28.57)	1 (7.69)	13 (36.11)	
Maghrebi	1 (2.04)	1 (7.69)	0 (0.00)	
Smoking				0.7545
Non-smoker	27 (55.10)	7 (53.85)	20 (55.56)	
Smoker	11 (22.45)	4 (30.77)	7 (19.44)	
Ex-smoker	11 (22.45)	2 (15.38)	9 (25.00)	
Time between diagnosis and treatment, years	0.18 (0.11-2.04)	0.91 (0.15-3.33)	0.16 (0.10-1.72)	0.2004
Time of continuous treatment, years	7.62 (5.48 – 11.87)	6.96 (3.96 – 8.22)	9.155 (5.79 – 13.05)	0.04268
Nadir CD4+ count, cells/mm³	333 (248-531)	320 (153.25-437)	348.5 (249.5-570)	0.3358
CD4+ count, cells/mm³	877 (611-1141)	1000 (500-1200)	820.5 (615.5-1120)	0.9548
CD8+ count, cells/mm³	872 (697-1193)	700 (500-1400)	875 (698.5-1150.25)	0.9009
CD4/CD8 ratio	1.01 (0.80 -1.40)	1.1 (0.60-1.60)	1.01 (0.84-1.295)	0.9188
Total HIV DNA in PBMCs, copies/10^6^ cells	131.41 (62.31-345.16)	153.15 (94.71-331.64)	126.66 (53.93-347.15)	0.7658
US HIV RNA in PBMCs, copies/µg total RNA	112.05 (38.38-238.58)	105.1 (13.1-276)	119 (44.8-233.5)	0.7453
Total HIV DNA in rectal tissue, copies/10^6^ cells	231 (85.03 -461.75)	363.3 (257.1-614.2)	207 (83.7-274)	0.07225
US RNA/T DNA ratio	0.69 (0.29-1.635)	0.47 (0.175-1.785)	0.7 (0.34-1.635)	0.4067
CDC classification				0.1738
A1	11 (23.91)	3 (23.08)	8 (24.24)	
A2	21 (45.65)	6 (46.15)	15 (45.45)	
A3	5 (10.87)	1 (7.69)	4 (12.12)	
B2	3 (6.52)	0 (0.0)	3 (9.09)	
B3	2 (4.35)	0 (0.0)	2 (6.06)	
C1	1 (2.17)	0 (0.0)	1 (3.03)	
C3	3 (6.52)	3 (23.08)	0 (0.0)	
NA	3 (6.52)	0 (0.0)	3 (9.09)	
HBV status				0.2436
Immune	32 (65.31)	9 (69.23)	23 (63.89)	
Non-immune, not infected	11 (22.45)	1 (7.69)	10 (27.78)	
Isolated HBc Ab	4 (8.16)	2 (15.38)	2 (5.56)	
Cured Hepatitis B	2 (4.08)	1 (7.69)	1 (2.78)	
HCV status				1
Not Infected	45 (91.84)	12 (92.31)	33 (91.67)	
Recovered	4 (8.16)	1 (7.69)	3 (8.33)	
CMV status				/
Positive	49 (100)	13 (100)	36 (100)	
Negative	0 (0)	0 (0)	0 (0)	

*^a^* Mann-Whitney tests were used for continuous variables and Fisher’s exact tests were used for categorical variables.

### 3.2 Associations between HIV persistence and inflammatory markers

**Figure 1:**
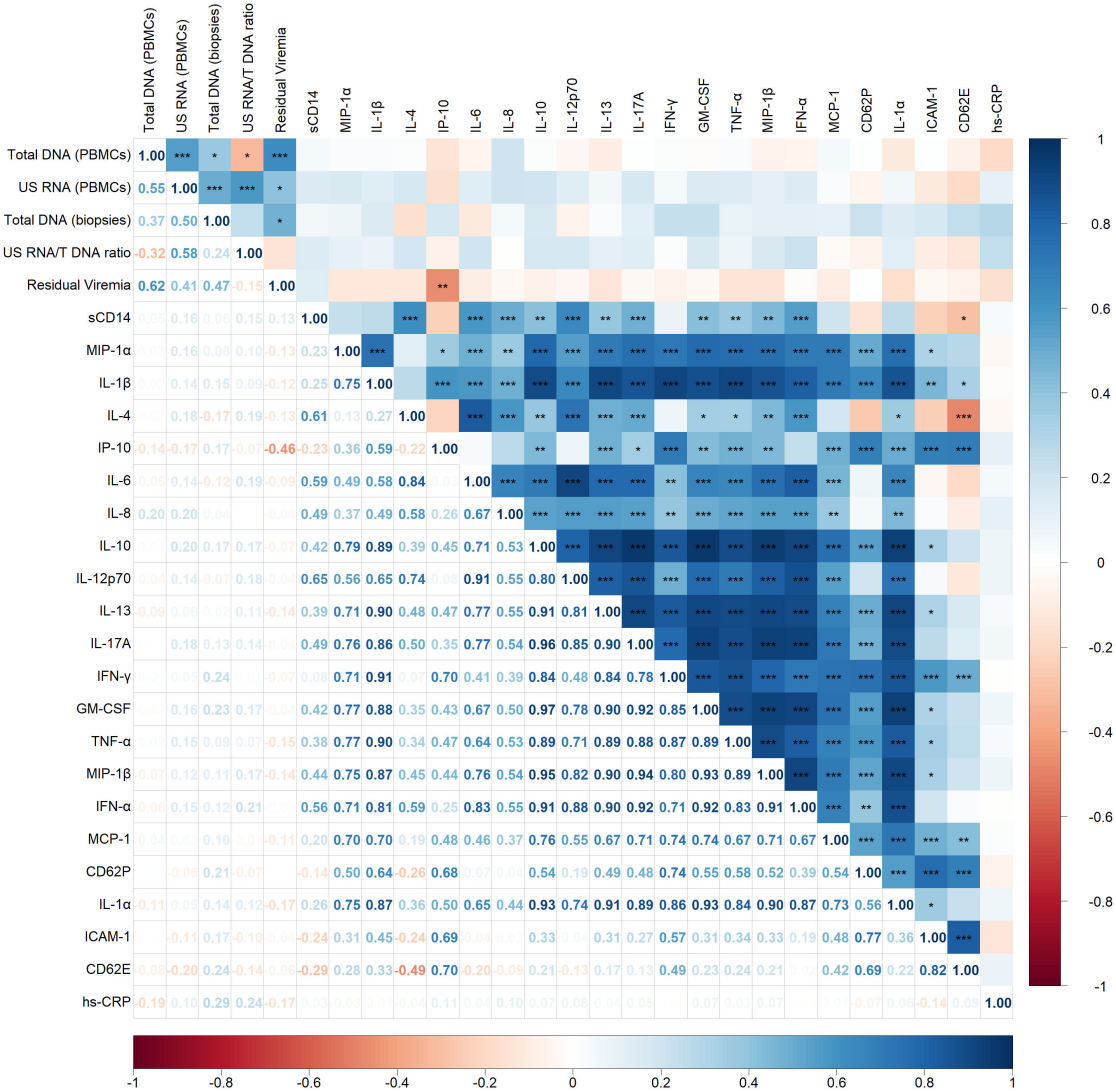
Spearman correlogram showing positive and negative correlations between HIV persistence and inflammatory markers. A heat map is used to indicate the strengths of associations between parameters with red representing negative correlations and blue representing positive correlations. The magnitude of the correlation coefficient, *rho*, indicates the degree of association, where values between 0.9 and 1.0 indicate very strong correlation, 0.7 to 0.9 indicate strong correlation, 0.5 to 0.7 indicate moderate correlation, 0.3 to 0.5 indicate weak correlation, and values below 0.3 indicate little to no linear correlation. Statistically significant correlations are marked with stars, where one star indicates *p* < 0.05, two stars indicate *p* < 0.01, and three stars indicate *p* < 0.001.

The Spearman correlogram in **Figure 1** demonstrates correlations between HIV persistence markers—including total HIV DNA (in PBMCs and rectal biopsies), unspliced (US) RNA (in PBMCs), and residual viremia—and various inflammatory markers.

Moderate to strong positive correlations were observed among the HIV persistence markers. Specifically, total DNA in PBMCs correlated positively with US RNA (p < 0.001, *rho* = 0.55) and residual viremia (p < 0.001, *rho* = 0.62). A weak positive correlation was also noted between total DNA in PBMCs and in biopsies (p < 0.05, *rho* = 0.37). US RNA correlated positively with total DNA in biopsies (p < 0.001, *rho* = 0.50) and demonstrated a positive correlation with residual viremia (p < 0.05, *rho* = 0.41). Additionally, total DNA in biopsies positively correlated with residual viremia (p < 0.05, *rho* = 0.47).

Surprisingly, apart from a negative correlation observed between residual viremia and IP-10 (CXCL10) (p < 0.01, *rho* = -0.46), no other significant correlations were identified between virological and inflammatory markers. Most inflammatory markers displayed strong positive correlations with each other, reflecting a highly interconnected inflammatory network. For instance, sCD14, a marker of monocyte activation, exhibited strong positive correlations with several cytokines, including IL-4 (p < 0.001, *rho* = 0.61), IL-6 (p < 0.001, *rho* = 0.59), IL-8 (p < 0.001, *rho* = 0.49), IL-12p70 (p < 0.01, *rho* = 0.65), IL-17A (p < 0.01, *rho* = 0.49) and IFN-α (p < 0.001, *rho* = 0.56).

Regarding other inflammatory interactions, there was a negative correlation between IL-4 and CD62E (p < 0.001, *rho* = -0.49), indicating an inverse relationship between this anti-inflammatory cytokine and this endothelial cell activation marker.

### 3.3 Associations between HIV persistence markers and immune cell populations

**Figure 2:**
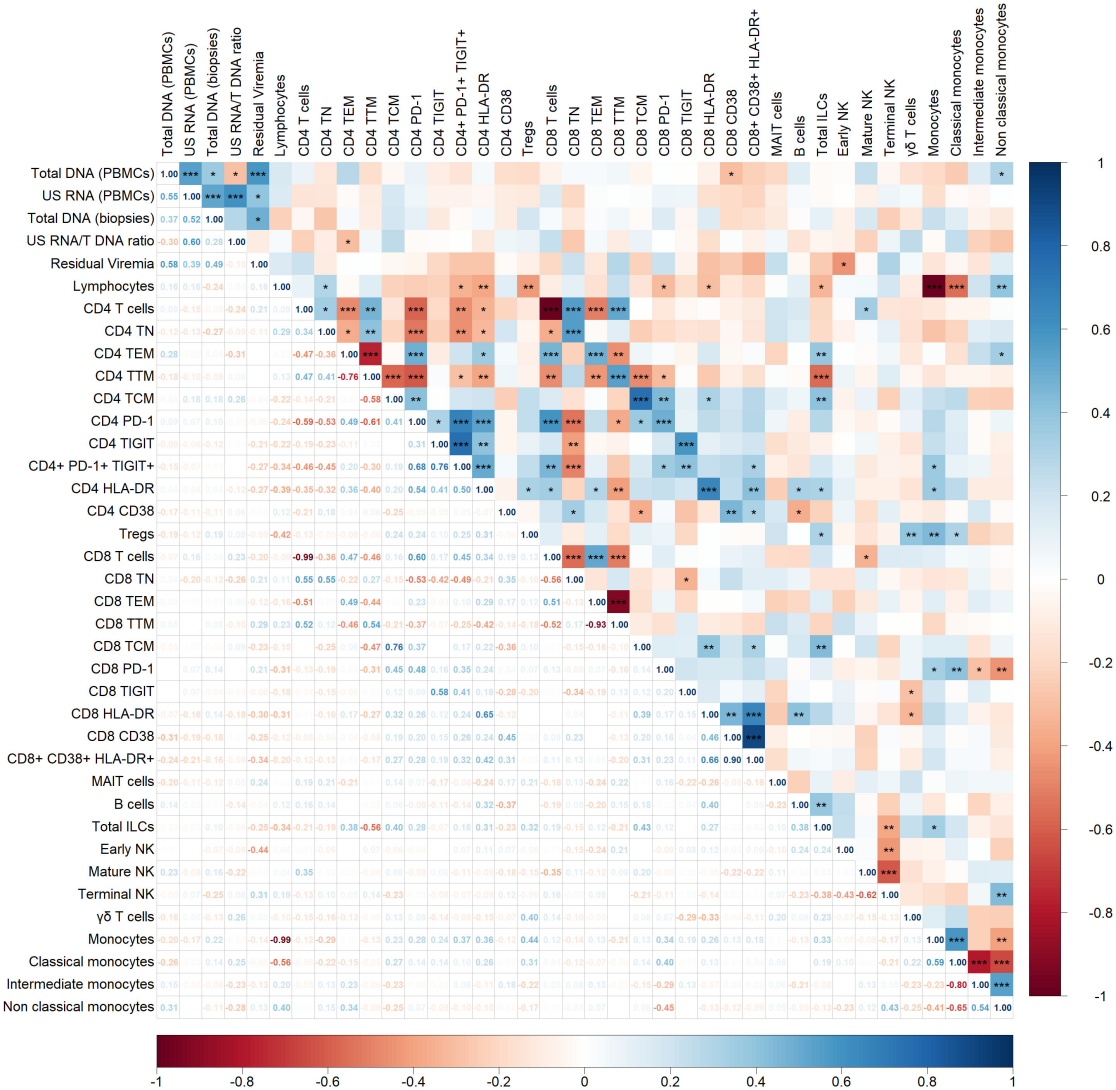
Spearman correlogram showing positive and negative correlations between HIV persistence markers and immune cell populations. A heat map is used to indicate the strengths of associations between parameters with red representing negative correlations and blue representing positive correlations. The magnitude of the correlation coefficient, *rho*, indicates the degree of association, where values between 0.9 and 1.0 indicate very strong correlation, 0.7 to 0.9 indicate strong correlation, 0.5 to 0.7 indicate moderate correlation, 0.3 to 0.5 indicate weak correlation, and values below 0.3 indicate little to no linear correlation. Statistically significant correlations are marked with stars, where one star indicates *p* < 0.05, two stars indicate *p* < 0.01, and three stars indicate *p* < 0.001.

Next, we examined correlations between HIV persistence markers and various immune cell populations including CD4 and CD8 T cells, B cells, monocytes and NK cells (**Figure 2**).

Total DNA in PBMCs showed a statistically significant but weak negative correlation with CD8+ T cells expressing the activation marker CD38 (p < 0.05, *rho* = -0.31). Additionally, total DNA positively correlated with non-classical monocytes (p < 0.05, *rho* = 0.31). The US RNA/Total DNA ratio negatively correlated with CD4+ effector memory T cells (p < 0.05, *rho* = -0.31). Residual viremia demonstrated a negative correlation with early NK cell populations (p < 0.05, *rho* = -0.44). No other correlation or trend was observed between HIV persistence markers and immune cell populations in our cohort.

Within immune cell subsets, several significant correlations were observed. Focusing on a statistically strong correlation with p < 0.001, positive correlations were noted within T cell subsets. For example, CD4+ T naïve cells positively correlated with CD8+ T naïve cells (*rho* = 0.55), and similar patterns were observed across effector memory (*rho* = 0.49), transitional memory (*rho* = 0.54), and central memory (*rho* = 0.76) subsets.

Immune exhaustion and activation markers also displayed positive correlations between CD4+ and CD8+ T cell subsets. For instance, PD-1 expression on CD4+ T cells correlated strongly with PD-1 expression on CD8+ T cells (*rho* = 0.48), and similar correlations were observed for TIGIT (*rho* = 0.58) and HLA-DR (*rho* = 0.65).

Finally, a correlation was observed among monocyte subsets, including classical, intermediate, and non-classical monocytes.

### 3.4 Associations between HIV persistence markers and clinical factors

**Figure 3:**
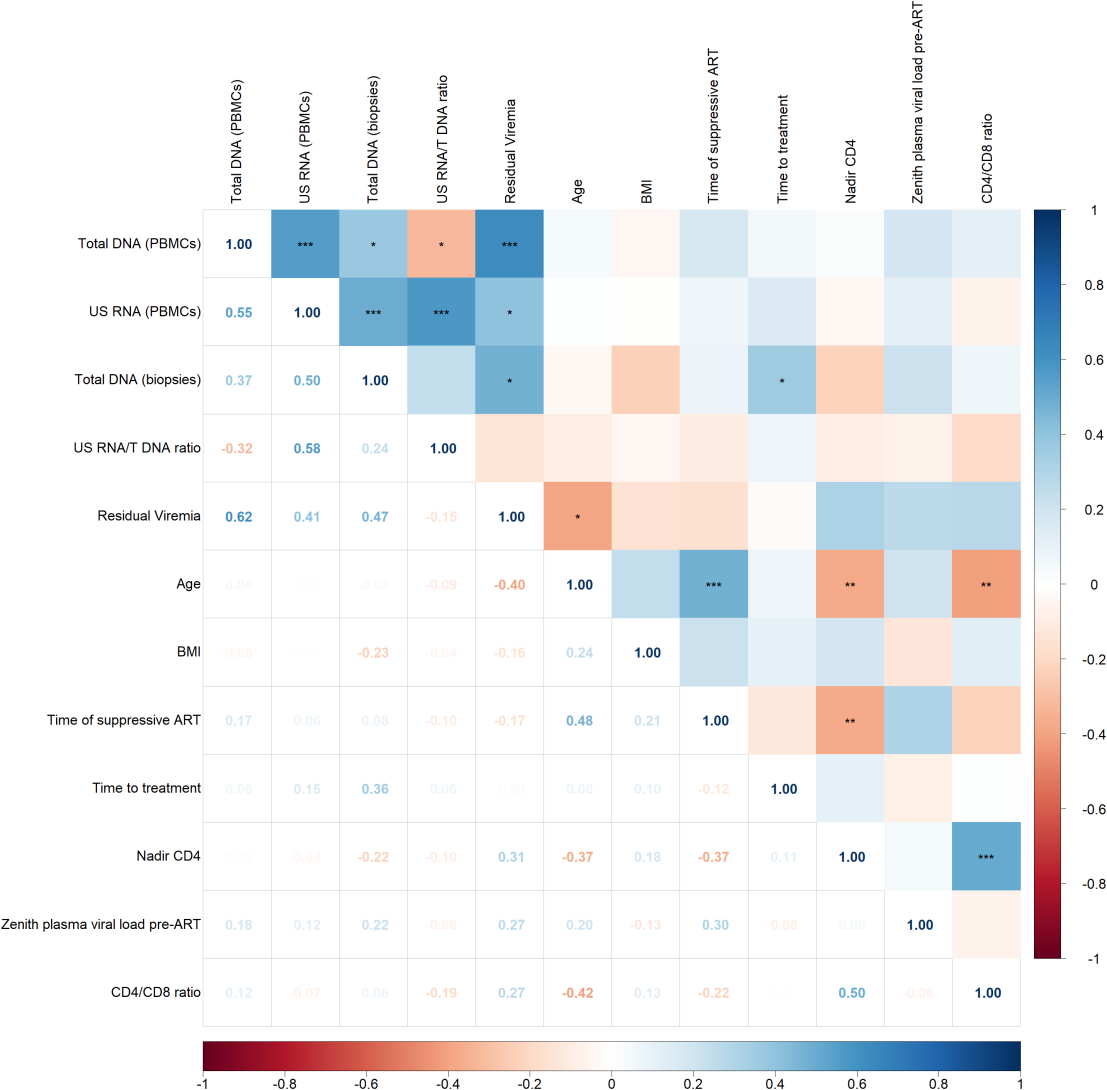
Spearman correlogram showing positive and negative correlations between HIV persistence markers and clinical factors. A heat map is used to indicate the strengths of associations between parameters with red representing negative correlations and blue representing positive correlations. The magnitude of the correlation coefficient, *rho*, indicates the degree of association, where values between 0.9 and 1.0 indicate very strong correlation, 0.7 to 0.9 indicate strong correlation, 0.5 to 0.7 indicate moderate correlation, 0.3 to 0.5 indicate weak correlation, and values below 0.3 indicate little to no linear correlation. Statistically significant correlations are marked with stars, where one star indicates *p* < 0.05, two stars indicate *p* < 0.01, and three stars indicate *p* < 0.001.

**Figure 3** illustrates the associations between HIV persistence markers and various clinical factors, including demographic characteristics (age and BMI), and parameters related to infection history and treatment (nadir CD4, CD4/CD8 ratio, zenith plasma viral load pre-ART, duration of suppressive ART, time to treatment initiation).

The correlogram reveals some statistically significant but weak correlations. Total HIV DNA in biopsies positively correlated with time on treatment (p < 0.05, *rho* = 0.36) and residual viremia negatively correlated with age (p < 0.05, *rho* = -0.40). No other significant correlations were observed between HIV persistence markers and clinical indicators.

Among clinical indicators and focusing on statistically strong correlations with p < 0.001, a significant positive correlation was found between nadir CD4 count and CD4/CD8 ratio (p < 0.001, *rho* = 0.50). Additionally, a positive correlation was observed between age and the duration of suppressive ART (p < 0.001, *rho* = 0.48). This reflects the expected trend that older individuals have been on suppressive ART for a longer period compared to younger individuals.

## 4 Discussion

In this study, we investigated the associations between HIV persistence markers, inflammatory markers, immune cell populations and clinical indicators in a cohort of 49 PWH on long-term DTG-based ART. The relationship between HIV persistence and immune activation has been a subject of debate, with the literature presenting conflicting findings.

Understanding the origins of persistent inflammation and immune activation in ART-treated PWH holds significant clinical implications. If inflammation is primarily driven by the HIV reservoir, therapeutic strategies should focus on reducing reservoir size or silencing its activity, for example, by inhibiting viral transcription (e.g., Tat inhibitors) or minimizing residual viral replication. Conversely, if the link between reservoirs and inflammation is weak, alternative drivers such as lifestyle factors (e.g., smoking, obesity), microbial translocation, co-infections, or other comorbidities must be prioritized. Identifying these drivers is essential for optimizing therapeutic interventions and improving long-term outcomes.

On the one hand, a significant portion of the literature supports a strong link between viral reservoirs and immune activation, suggesting an interdependent relationship between HIV persistence and inflammation (13). Early evidence from Chomont et al. demonstrated that HIV proviral DNA preferentially resides in CD4+ T cells expressing immune activation (PD-1) and proliferation (Ki67) markers (31). Further analyses across different compartments – blood, rectal tissue and lymph nodes – confirmed a positive correlation between the frequency of PD-1+ CD4+ T cells in blood and total HIV DNA, as well as between PD-1+ CD4+ T cells in rectal tissue and both integrated HIV DNA and CA US HIV RNA (32). Similarly, strong positive associations were observed in rectal tissue and lymph nodes between CD8+ T cells expressing HLA-DR and CD38 and both integrated HIV DNA and CA US HIV RNA. Later, Hatano et al. corroborated these findings, demonstrating significant associations between proviral DNA levels and the frequency of PD-1-expressing CD4+ T cells in blood samples. They further concluded that low CD4+ T cell count (<350 cells/mm^3^), despite suppressive therapy, was linked to higher CA RNA levels, proviral DNA levels and increased frequencies of CD4+ T cells expressing CD38, HLA-DR, CCR5 and/or PD-1 (33). The same group found a consistent association between CD4+ and CD8+ T cells expressing HLA-DR and the frequency of resting CD4+ T cells containing HIV DNA (34). In a more recent study conducted in 2021 by Olson et al., the focus shifted from total or integrated HIV DNA and CA RNA to intact proviral DNA (35). Interestingly, while intact proviral DNA did not correlate with plasma inflammation markers (e.g. D-dimer), a significant correlation was observed with intracellular HIV RNA. This finding suggests that the viral transcriptional activity, rather than the presence of intact DNA, may play a more important role in driving inflammation in ART-treated individuals. Furthermore, the study linked aging to reduced control over HIV-1 transcription, highlighting how age-related immune changes may exacerbate viral transcription and its inflammatory consequences in long-term ART-treated individuals. In line with these results, Scherpenisse et al. demonstrated that HIV-1 transcription at an early time point during ART correlates with markers of immune activation, exhaustion, and apoptosis (36).

Another critical marker of HIV persistence, low-level residual viremia, has been associated with microbial translocation and systemic inflammation (37–39). Riddler et al. found that residual viremia was associated with higher CD8+ T cell counts and lower CD4/CD8 ratio in ART-treated individuals, suggesting incomplete immune recovery (40). In a study by Falasca et al., residual viremia showed a significant correlation with sCD14 levels, an established marker of microbial translocation and monocyte activation (41). However, this study found no correlations between HIV DNA, HIV RNA, and inflammatory markers, highlighting inconsistencies with previous findings. Finally, several studies on raltegravir intensification have reported reductions in HIV replication markers, such as 2-LTR circles or US HIV RNA, along with decreased immune activation levels (42–49).

On the other hand, some intensification studies have failed to replicate these findings (50–56) and several studies have not consistently found a strong relationship between HIV persistence markers and immune activation (35, 57–63). For example, Gandhi et al. proposed that inflammation and immune activation in ART-treated individuals might predominantly come from immunologic events occurring before ART initiation rather than being perpetuated by ongoing viral replication sustained by persistent low-level viremia during suppressive ART (63). Recently, Bailon et al. demonstrated that a larger reservoir size was initially associated with higher levels of soluble inflammatory biomarkers, as well as increased activation and exhaustion of CD4+ and CD8+ T cells (64). However, these associations disappeared upon achieving viral suppression, regardless of the antiretroviral regimen used (dual or triple therapy). Similarly, previous comparative studies between two-drug regimens (2DRs) and three-drug regimens (3DRs) have not shown consistent differences in inflammatory markers, residual viremia or T-cell activation and exhaustion markers (65–68).

Overall, our findings revealed limited association between HIV persistence markers – including both blood and tissue reservoirs – and systemic inflammation or immune activation. However, some of our observations aligned with previous research. Notably, we observed an association between NK cells and residual viremia, a relationship that has been documented in both adults and children (69–72). This finding underscores the pivotal role of NK cells as key effectors of the innate immune system, highlighting their potential contribution to the control and modulation of HIV reservoir cell dynamics.

Several factors could explain the lack of a strong link between HIV persistence and inflammation in our study. First, compared to earlier studies from Chomont and Hatano, our cohort exhibited relatively low reservoir sizes, with a median total HIV DNA of 131 copies/10^6^ cells in PBMCs and 231 copies/10^6^ cells in rectal tissue (31, 33). However, in comparison to the LoViret cohort described by Galvez et al., which reported exceptionally low reservoir levels in chronically treated HIV-1 infected individuals (<50 HIV-DNA copies/10^6^ PBMC), the reservoir sizes in our cohort were comparatively higher (73). Reservoir activity, as measured by unspliced RNA levels, was also limited (median: 112 copies/µg total RNA). Although direct comparison with earlier studies is challenging due to differences in sampling methods (e.g., CD4-enriched populations vs. PBMCs) and techniques, these values are in line with limited reservoir size and activity in these participants. However, in comparison to a recent study of early treated adolescents living with HIV and receiving long-term suppressive ART, our cohort exhibited higher levels of CA US HIV RNA, as that study reported a median of 19 copies/10^6^ PBMCs (74).

These discrepancies likely reflect key changes in the management of PWH, including the initiation of treatment regardless of CD4+ T cell count and advancements in ART regimens, particularly the widespread adoption of integrase inhibitor-based ART (75). The impact of these modifications in PWH care on HIV reservoir size and activity should not be underestimated. In recent cohorts, these changes have resulted in prolonged viral suppression, such as ours (median duration: 7.62 years), and treatment with more potent regimens such as the DTG-based regimen that was used by all participants of the present study.

Longitudinal studies evaluating the dynamics of HIV reservoir cells and the impact of the host immune system have recently emerged, alongside new technologies like the intact proviral DNA assay (IPDA) and near full-length individual proviral next-generation sequencing. These PCR-based methods have revealed significant differences in the longitudinal dynamics of intact versus defective proviruses. Intact proviruses decline more rapidly, likely due to the active immunological elimination of transcriptionally active reservoirs, which are more easily recognized and targeted by the immune system (76). Defective proviruses that encode proteins also exhibit a shorter half-life compared to those incapable of protein expression (77, 78). Over time, the HIV reservoir is thus progressively reshaped into a more silent and defective state under the persistent pressure of the immune system, which preferentially targets infected cells with higher residual proviral transcriptional activity (79).

We thus hypothesize that, in virally suppressed individuals, the contribution of the HIV reservoir to chronic immune activation and inflammation will diminish over time on suppressive ART.

In our cohort, this is likely reflected by a reservoir dominated by deeply latent or transcriptionally silent clones which contribute minimally to inflammation and immune activation. This aligns with the prolonged duration of viral suppression and the robust immune recovery observed in our participants (median CD4+ counts: 877 cells/mm^3^ and CD4/CD8 ratio ~ 1). In addition, all participants in this study were receiving an integrase inhibitor-based regimen. Integrase inhibitor-based therapies are known to achieve faster and probably stronger viral suppression compared to protease inhibitor or non-nucleoside reverse transcriptase inhibitor-based regimens (80–82).

These findings reinforce the idea that inflammation and immune activation in ART-treated PWH are influenced by a multifaceted interplay of factors extending beyond HIV persistence alone. Moreover, they suggest that the contribution of the viral reservoir to inflammation may vary substantially between individuals and even in the same individuals over time, depending on the clonal dynamics that dominate the proviral landscape. Such variability highlights the importance of considering both host and viral factors when evaluating the drivers of inflammation in ART-treated populations.

While our findings are informative, several limitations should be acknowledged. First, the modest sample size (n=49) may have limited statistical power to detect subtle correlations. Second, we did not evaluate the impact of common sexually transmitted infections (e.g., chlamydia, gonorrhoea, syphilis) or examine gut-microbiome dysbiosis in detail, even though emerging evidence links microbiome alterations to chronic inflammation and non-communicable diseases (83, 84). Third, cell-associated unspliced HIV RNA could not be measured reliably in rectal tissue. Fourth, key immunological features such as immune-senescence markers and the Th17/Treg balance were not assessed. Taken together, these constraints should be borne in mind when interpreting the study’s conclusions.

In conclusion, the narrative surrounding HIV persistence and its impact on inflammation has shifted. Earlier studies described a scenario where larger, more active reservoirs perpetuated immune activation and inflammation. In contrast, today’s PWH, treated earlier with more potent regimens, have smaller, less active reservoirs that exert a diminished impact on immune activation. This highlights the dynamic nature of reservoir evolution under long-term ART and underscores the importance of considering these shifts when interpreting contemporary findings.

## Data Availability

The original contributions presented in the study are included in the article/**Supplementary Material**. Further inquiries can be directed to the corresponding authors.
