# Supplementary material for: No associations between HIV reservoir and inflammation in long-term virally suppressed dolutegravir-based ART-treated individuals
Source: Front Immunol. 2025 Jul 28;16:1628086. doi: 10.3389/fimmu.2025.1628086 (PMC12336182; doi:10.3389/fimmu.2025.1628086)
Supplement: Supplementary file 1 [file Table1.docx]

**Supplementary tables**

**Table 2.** Virological and immunological data of participants at baseline.

|  | All  (n=49) | EDIT  (n=13) | IDOLTIB  (n=36) | p^[[1]](#endnote-1)^ |
| --- | --- | --- | --- | --- |
| **HIV latent reservoir** |  |  |  |  |
| Total HIV DNA in PBMCs, copies/10^6^ cells | 131.41 (62.3075-345.1575) | 153.15 (94.71-331.64) | 126.66 (53.925-347.145) | 0.7658 |
| US HIV RNA in PBMCs, copies/µg total RNA | 112.05 (38.375-238.575) | 105.1 (13.1-276) | 119 (44.8-233.5) | 0.7453 |
| Total HIV DNA in rectal tissue, copies/10^6^ cells | 231 (85.025-461.75) | 363.3 (257.1-614.2) | 207 (83.7-274) | 0.07225 |
| US RNA/ T DNA ratio | 0.69 (0.29-1.635) | 0.47 (0.175-1.785) | 0.7 (0.34-1.635) | 0.4067 |
| Residual viremia, HIV RNA copies/mL | 1.31 (0.765-2.47) | / | 1.31 (0.765-2.47) | / |
| Zenith plasma viral load pre-ART, HIV RNA copies/mL | 53900 (19953-130000) | 85350 (18775-168000) | 47600 (24200-127000) | 0.8747 |
| **Inflammation** |  |  |  |  |
| sCD14 (ng/mL) | 840.8 (650.79 -1128.22) | 678.9 (588.4-760.2) | 949.315 (740.2275-1242.45) | 0.004547 |
| MIP-1 alpha (pg/mL) | 4.81 (2.75-7.19) | 7.23 (5.09-10.5) | 4.38 (2.4925-5.4475) | 0.014 |
| IL-1 beta (pg/mL) | 23 (2.1-39.5) | 37.5 (27.8-65.4) | 16.36 (0.915-33.615) | 0.006346 |
| IL-4 (pg/mL) | 21.83 (16.8-28.12) | 7.2 (5.9-8.6) | 23.915 (21.34-30.9575) | 0.000001184 |
| IP-10 (pg/mL) | 5.73 (4.68-7.3) | 9.2 (7.3-10.7) | 5.235 (4.59-5.89) | 0.000006581 |
| IL-6 (pg/mL) | 35.84 (24.25-78.75) | 26.6 (15.98-35.84) | 50.93 (30.75-105.0525) | 0.005925 |
| IL-8 (pg/mL) | 1.3 (0.88-2.34) | 1.14 (0.8-1.43) | 1.375 (1.0175-2.3625) | 0.2129 |
| IL-10 (pg/mL) | 4.83 (2.45-8.04) | 5.6 (4.7-10.7) | 4.025 (2.29-7.455) | 0.08319 |
| IL-12p70 (pg/mL) | 77.59 (44.6-152.59) | 56.8 (43.5-63.7) | 97.835 (47.3375-179.2625) | 0.06493 |
| IL-13 (pg/mL) | 10 (5.82-15.97) | 13.6 (8.9-15.8) | 9.055 (5.6675-16.0975) | 0.2171 |
| IL-17A (pg/mL) | 19.7 (11.68-29.6) | 24.3 (18.1-28.1) | 18.905 (10.93-31.43) | 0.371 |
| IFN gamma (pg/mL) | 22 (12.55-39.96) | 43.3 (33.9-52.3) | 15.065 (11.4725-22.59) | 0.00002401 |
| GM-CSF (pg/mL) | 103.82 (57.03-153.7) | 142.1 (83.4-198.9) | 81.885 (41.5-140.32) | 0.0501 |
| TNF alpha (pg/mL) | 38.06 (22.25-53.3) | 44.7 (37.7-53.3) | 28.085 (20.23-43.575) | 0.0242 |
| MIP-1 beta (pg/mL) | 38.57 (26.5-48.6) | 40.6 (37.5-45.7) | 32.44 (25.23-51.625) | 0.2344 |
| IFN alpha (pg/mL) | 2.2 (1.28-3.5) | 2.5 (1.3-2.9) | 2.08 (1.245-3.6575) | 0.9368 |
| MCP-1 (pg/mL) | 21.15 (16.46-26.41) | 26 (22.8-31.3) | 19.685 (14.615-24.96) | 0.02151 |
| CD62P (pg/mL) | 28255.31 (16423.24-46525.55) | 66712.3 (51149.6-72684.8) | 21052.82 (11980.94-36660.02) | 0.0000004162 |
| IL-1 alpha (pg/mL) | 1.68 (1.2-2.5) | 2.2 (1.5-3.1) | 1.49 (1.1075-2.3275) | 0.04879 |
| ICAM-1 (pg/mL) | 24400.01 (18273-36180.9) | 39746.1 (31957.6-52317.5) | 20077.88 (15302.73-27914.9) | 0.000008274 |
| CD62E (pg/mL) | 9605.17 (6564.91-14244.9) | 17142.4 (14884.6-20310) | 7449.93 (6391.203-10196.31) | 0.000000002841 |
| hsCRP (mg/L) | 2.07 (0.95-3.35) | 2.3 (1-2.9) | 1.945 (0.85-3.6175) | 0.6752 |
| Immune activation | N = 46 | N = 13 | N = 33 |  |
| PD-1+ in CD4 T cells (%) | 17.2 (11.15-21.725) | 20.5 (18+23.3) | 14.9 (9.42-20) | 0.01917 |
| TIGIT+ in CD4 T cells (%) | 21.45 (16.175-26.9) | 17.2 (15.7-21) | 24.8 (18.2-27.9) | 0.08319 |
| HLA-DR+ in CD4 T cells (%) | 3.705 (2.4825-4.8525) | 4.37 (3.81-6.84) | 2.81(2.06-4.19) | 0.007551 |
| CD38+ in CD4 T cells (%) | 2.98 (2.1425-4.85) | 2.42 (1.32-4.87) | 3.2 (2.68-4.79) | 0.1399 |
| PD-1+ in CD8 T cells (%) | 21.7 (14.925-27.075) | 24.2 (15.4-35.1) | 20.8 (14.9-26.4) | 0.3113 |
| TIGIT+ in CD8 T cells (%) | 43.85 (32.325-51.825) | 35.2 (31.8-46.4) | 46.8 (35.5-57.4) | 0.1607 |
| HLA-DR+ in CD8 T cells (%) | 4.07 (2.5675-7.4025) | 9 (6.38-14.9) | 3.18 (2.23-5.24) | 0.0004038 |
| CD38+ in CD8 T cells (%) | 1.74 (1.21-2.6325) | 1.87 (1.33-2.69) | 1.61 (1.2-2.46) | 0.9319 |

*i* Mann-Whitney tests were used for continuous variables.

**Table 3.** List of monoclonal antibodies.

| Specificity | Clone | Fluorophores |
| --- | --- | --- |
| CD45RA | HI100 | BV510 |
| CD28 | CD28.2 | BV750 |
| CCR7 | 2-L1-A/G043H7 | BV650 |
| CD56 | HCD56 | BV570 |
| CD127 | A019D5 | BV785 |
| TCRVα7.2 | 3C10 | BV605 |
| TIGIT | 741182/A15153G | BV421 |
| CD38 | HIT2 | BV711 |
| CD20 | 2H7 | Pac blue |
| CD8 | HIT8a | PercP |
| CD19 | SJ25C1 | BB700 |
| CD14 | 63D3 | Spark blue 550 |
| CD11c | 3.9 | PerCP-efluor710 |
| HLA-DR | G46-6 | BB515 |
| CD45 | HI30 | Spark YG 593 |
| TCRγδ | B1 | PE-Cy7 |
| IgD | IA6-2 | PE-Cy5 |
| CD25 | M-A251 | PE-Dazzle594 |
| CD16 | 3G8 | PE-Fire 640 |
| PD-1 | EH12.1/EH12.2H7 | PE |
| CD4 | SK3 | PE/Fire 700 |
| CD3 | SK7 | APC/Fire 810 |
| CD27 | L128/QA17A18 | R718 |
| CD123 | S18016F | Spark nir 685 |
| LAG-3 | T47-530/11C3C65 | AF647 |
| live/dead |  | Zombie NIR |

**Supplemental data 1**

Inclusion and exclusion criteria for the EDIT study

Inclusion criteria

- Men or women of at least 18 years of age with a confirmed HIV infection.
- The patient must have been on ART for more than 24 months with stable treatment for at least
- 6 months and a viral load consistently below 20 copies/mL during the 12 months prior to screening.
- The patient should have a CD4+ T lymphocyte count above 200 cells/mm3.

Exclusion criteria

- Patient suffering from active viral hepatitis or under treatment for hepatitis B or C.
- Patient suffering from a tumour that may require chemotherapy or radiotherapy.
- Patient suffering from hepatic (Child-Pugh score A, B, or C) or renal (estimated glomerular
- filtration rate <50 mL/min) failure.
- Patient suffering from a gastrointestinal pathology that may affect the absorption of
- dolutegravir (bypass, short hail, ...).
- Patient suffering from thrombocytopenia, coagulation disorder or medication that may cause
- bleeding (treatment with antiplatelet drugs or anticoagulants).
- Patient taking drug treatment responsible for significant interaction with dolutegravir
- according to the University of Liverpool website (https://www.hiv-druginteractions.org/).
- Pregnancy or breastfeeding.
- Any acute pathology within 8 weeks prior to inclusion.
- Any condition that, in the judgement of the investigators, could compromise the patient's
- adherence to the protocol.
- Patient already on dolutegravir twice daily or other integrase inhibitor therapy (elvitegravir or
- raltegravir).
- Patient with rectoanal pathology that contraindicates biopsy (e.g., anal stenosis).

**Supplemental data 2**

Inclusion and exclusion criteria for the IDOLTIB study

Inclusion Criteria

- Male or female, aged 18 years or older, infected with HIV-1.
- The patient must have been on ART for more than 24 months with a stable regimen of DTG+3TC+ABC for at least 6 months, and a consistently undetectable viral load (less than 20 copies/ml) during the 24 months before screening.
- The patient must have a CD4 lymphocyte count greater than 200 cells/mm³.

Exclusion Criteria

- Patient with chronic replicative viral hepatitis or undergoing treatment for hepatitis B or C.
- Patient with a tumor that may require chemotherapy or radiotherapy.
- Patient with hepatic insufficiency (Child-Pugh Score A, B, or C) or renal insufficiency (GFR < 50 ml/min).
- Patient with a gastrointestinal condition that could affect the absorption of DTG or lamivudine (e.g., gastric bypass, short bowel syndrome, etc.).
- Pregnant or breastfeeding woman.
- Patient with thrombocytopenia, a coagulation disorder, or on medications that increase the risk of bleeding (e.g., antiplatelet or anticoagulant therapy).
- Any acute illness within 8 weeks prior to inclusion.
- Any condition that, in the investigators' judgment, could compromise the patient's adherence to the protocol.
- Patient on a twice-daily DTG regimen or on another integrase inhibitor (Elvitegravir or Raltegravir).
- Patient with a recto-anal condition contraindicating biopsy (e.g., anal stenosis).
- Patient with active tuberculosis.
- Known resistance to DTG or 3TC.

1. [↑](#endnote-ref-1)
